# RPI-Pred: predicting ncRNA-protein interaction using sequence and structural information

**DOI:** 10.1093/nar/gkv020

**Published:** 2015-01-21

**Authors:** V. Suresh, Liang Liu, Donald Adjeroh, Xiaobo Zhou

**Affiliations:** 1 Department of Radiology, Wake Forest University Health Science, Medical Center Boulevard, Winston-Salem, NC 27157, USA; 2 Lane Department of Computer Science and Electrical Engineering, West Virginia University, Morgantown, WV 26505, USA

## Abstract

RNA-protein complexes are essential in mediating important fundamental cellular processes, such as transport and localization. In particular, ncRNA-protein interactions play an important role in post-transcriptional gene regulation like mRNA localization, mRNA stabilization, poly-adenylation, splicing and translation. The experimental methods to solve RNA-protein interaction prediction problem remain expensive and time-consuming. Here, we present the RPI-Pred (RNA-protein interaction predictor), a new support-vector machine-based method, to predict protein-RNA interaction pairs, based on both the sequences and structures. The results show that RPI-Pred can correctly predict RNA-protein interaction pairs with ∼94% prediction accuracy when using sequence and experimentally determined protein and RNA structures, and with ∼83% when using sequences and predicted protein and RNA structures. Further, our proposed method RPI-Pred was superior to other existing ones by predicting more experimentally validated ncRNA-protein interaction pairs from different organisms. Motivated by the improved performance of RPI-Pred, we further applied our method for reliable construction of ncRNA-protein interaction networks. The RPI-Pred is publicly available at: http://ctsb.is.wfubmc.edu/projects/rpi-pred.

## INTRODUCTION

RNA-protein interactions (RPI) play a crucial role in fundamental cellular processes, such as human diseases (1), viral replication and transcription (2,3) and pathogen resistance in plants (4–6). Recent high-throughput techniques produce remarkable evidences to prove that protein can interact with RNA to mediate different kinds of cellular functions. During the post-transcriptional regulation process, RPI complex interacts with targeted mRNAs and/or non-coding RNAs (ncRNAs) to regulate cellular functions, such as RNA splicing, RNA transport, RNA stability and RNA translation (7–9). Experimental studies on RPI reveal that many functional ncRNAs play pivotal roles in gene expression and regulation (10–16). Although a few individual ncRNAs have been well studied, e.g. HOTAIR (17), MALAT-1 (18) and Xist (19), the majority are still not well understood. Over 30 000 ncRNAs have been identified and this number is expected to increase every year (14,15,20). Currently, NPInter (21) is the only database, which provides the functional information for all the experimentally validated ncRNA-protein interactions (ncRPI). The experimental techniques are generally time-consuming and expensive. Our understanding of function of individual ncRNAs is far outpaced by the sheer volume and diversity of the available data. Furthermore, our understanding of ncRPI in gene regulatory networks is very limited, especially when compared to the regulatory roles of protein–protein and DNA–protein complexes. This is because the advances in genomics and proteomics techniques have resulted in tremendous amounts of data on protein–protein and protein–DNA interactions (22–24); however, much less information is available on ncRPI.

In despite of the increasing amount (∼400) of successfully identified RNA binding proteins (RBP) in the human genome (25,26), we still lack a complete understanding of RPI complexes and their roles in post-transcriptional regulatory networks (7,27). Although the sequence-homology-based approaches, such as Basic Local Alignment Search Tool (28–30) and PFAM (31–33), helped in detecting the functional regions (binding domains) of proteins and therefore the possible functions, these approaches lack the ability to identify the interacting partners (RNAs) for a given protein, or determine whether a given pair of protein and RNA can form interaction or not. To our knowledge, currently very few computational approaches are available to predict RPIs. One of the first computational methods for predicting ncRPI was reported in 2011 by Pancaldi and Bähler (34). They trained random forest (RF) and support vector machine (SVM) classifiers using more than 100 features extracted from protein secondary structure and localization, protein and gene physical properties and untranslated regions (UTRs). Thereafter, catRAPID (35) was developed by exploiting the physicochemical properties including secondary structure, hydrogen bonding and van der Waals propensities. Next, Muppirala *et al.* (36) introduced a method called RPISeq, which was constructed by using the features derived from protein and RNA sequences. They also trained RF and SVM classifiers using 3-mer and 4-mer conjoint triad features for amino acid and nucleotide sequences, respectively (37). Wang *et al.* (38) proposed an approach based on Naïve Bayes (NB) and Extended NB (ENB) classifiers using the same data sets and similar triad features reported in Muppirala *et al.*'s work. More recently, Lu *et al.* (39) proposed a method called ‘lncPro’ for predicting ncRNA-protein associations, using Fisher linear discriminant approach. His training features were three types of classical protein secondary structures, hydrogen-bond and Van der Waals propensities, as well as six types of RNA secondary structures (RSS).

Muppirala *et al.* (36) and Wang *et al.* (38) proposed their methods based on sequence features to predict RPI interactions. Other methods (34,35) have also been proposed by combining sequence and structural features. lncPro (39) method used protein and RSS, hydrogen-bond and Van der Waals propensities. However, none of the above methods used the high-order 3D protein and RNA structure features, which are known to be the key of their possible functions (40).

In the present work, we presented a computational approach to predicting protein-RNA interaction pairs and/or identify the binding partners of a given protein or RNA from candidates. In addition to sequence features, we combine the high-order structures of both proteins and RNAs, for a comprehensive understanding of RPI interactions. We consider the protein structures in terms of 16 structural fragments called protein blocks (PBs) (41). The PBs provide a more accurate representation of known protein structures than classical three state protein secondary structures (α-helix, β-sheet and coil), and have been applied in many protein structure-based analysis (42,43). For the RNA high-order structure, we considered five classes of RSS, namely, stem, hairpin, loop, bulges and internal loop. These PB and RSS were combined with their corresponding amino acid and nucleotide sequences. Using these features, we developed a SVM-based machine learning approach, RPI-Pred, to predict protein-RNA interactions. Our training database was constructed using sequence and experimentally validated structures of proteins and RNAs from the Protein Data Bank (PDB) (44). We also used sequence and predicted structures to test our RPI-Pred on different data sets, such as RPI369 and RPI2241, and ncRPI data sets, such as RPI367, RPI13243 and NPInter10412 (21). We extended our analysis to construct an *in silico* network to study potential interactions between proteins and ncRNAs, which can help us in further understanding of ncRNA's functions. Finally, a web server for this proposed method was also developed and freely accessed at http://ctsb.is.wfubmc.edu/projects/rpi-pred.

## MATERIALS AND METHODS

### Work flow

**Figure 1.**
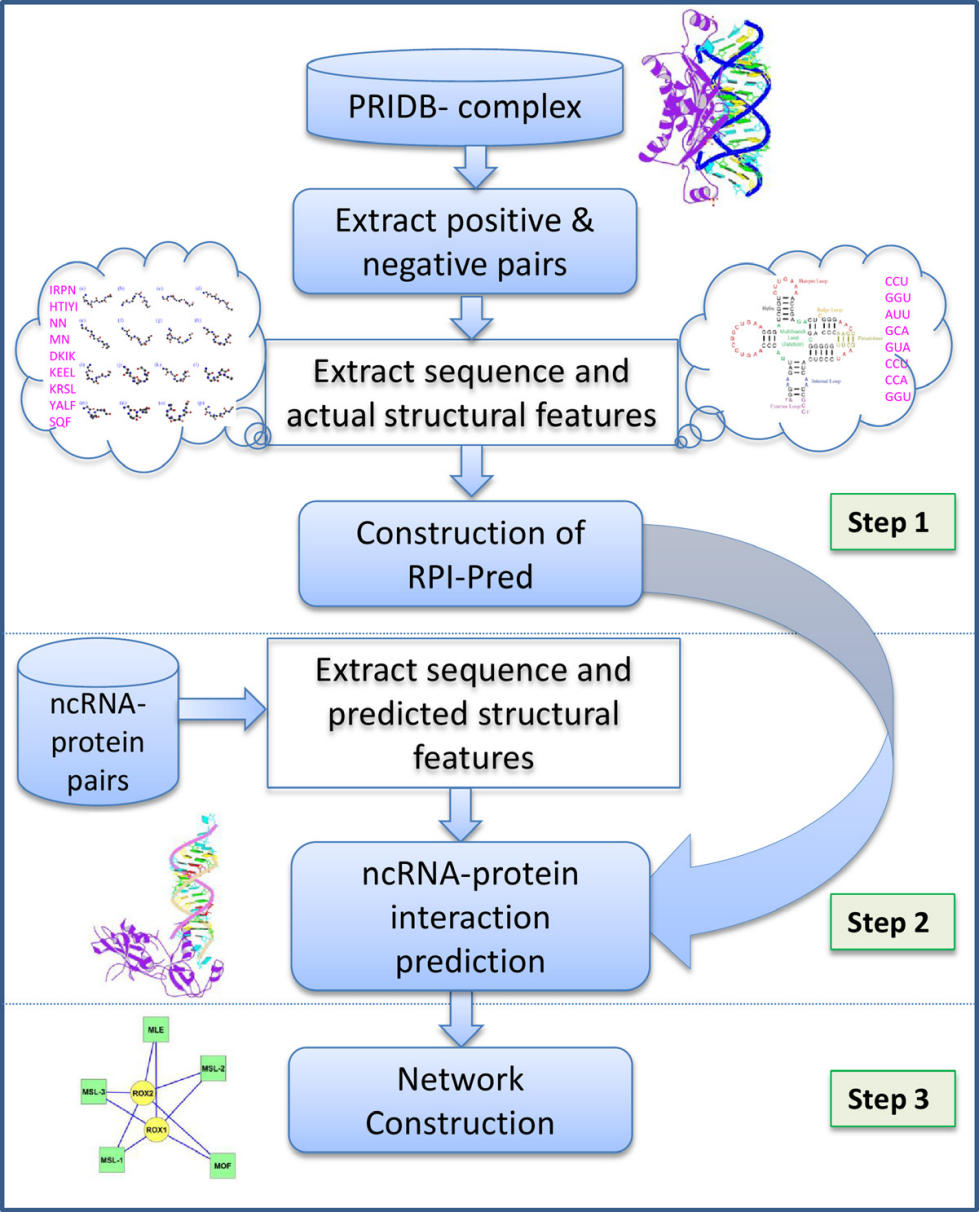
Step-wise work flow for the proposed RPI-Pred method.

Figure 1 shows the work flow for the development of RPI-Pred method. The proposed method includes three steps: (1) extraction of sequence and structure features for protein and RNA to develop the RPI-Pred prediction method, (2) prediction of ncRPI and (3) construction of the *in silico-*based biological network on the predicted results in step 2. Step 1 includes various processes, such as construction of the training data set, removal of redundant RNA-protein pairs, feature extraction from sequences and structures in the training data set and development of the ‘RPI-Pred’ model. Step 2 includes the feature extraction in terms of primary sequence and predicted structure for given protein and/or ncRNA and ncRPI prediction using RPI-Pred. Step 3 consists of construction of interaction networks based on RPI-Pred interaction predictions. More detailed descriptions for each step are given below.

### Training data sets

To develop the RPI-Pred method, first, we build a non-redundant training data set of RPI complexes by parsing the Nucleic Acid Database (NDB) (39) and the protein-RNA interface database (PRIDB) (45). The former provides data for RNA-protein complexes, whereas the latter provides atomic interfaces for RNA-protein interacting pairs. A total of 1560 RPI complexes available in NDB (as of 1 February 2014) were used in this study. We extracted the atomic and chain interfaces for 1336 complexes from PRIDB, resulting in 13 163 protein and 2715 RNA chains. These 1336 complexes were further used to construct our training data set, which consist of both possible positive and negative protein-RNA pairs.

The procedure for constructing the training data set, included removing redundant protein/RNA pairs through sequence similarity criteria, is as follows. For instance, the RPI complex with the PDB id ‘1a9n’ has four protein chains (A, B, C, D) and two RNA chains (Q, R), respectively. We obtained the possible interaction pairs from PRIDB as A-Q, B-Q, C-R and D-R. Then the homologous RNA-protein pairs (i.e. similar protein chains interacting with similar RNA chains) were removed by searching the sequence similarity between protein (RNA) sequences. In this study, we used EMBOSS needle program (46) with the standard sequence identity cut-off ≥30% to remove proteins (and RNA chains) with a high sequence similarity. In the current example with ‘1a9n’, the protein chains A&C, B&D and RNA chains Q&R are 100% sequence-similar, therefore, we removed the redundant protein pairs. Finally, A-Q and B-Q were identified as non-redundant RNA-protein pairs.

The above selected non-redundant pairs were further tested for atomic interactions with a distance threshold (3.40 Å). This distance threshold helped to strengthen positive pairs in the training data set by including only strongly interacting RPI pairs. Different thresholds have been used to distinguish the binding RNA-protein pairs from non-binding ones (35,36,38,39,47). We used the threshold 3.40 Å (47). The threshold 3.4 Å is reasonable and sufficient to cover ‘strong’ and ‘moderate’ hydrogen bonds and energy-rich van der Waals contacts (48). Therefore, we set the threshold (3.4 Å) to distinguish the strongly interacting protein-RNA pairs (positive pairs) and weakly interacting protein-RNA pairs (negative pairs). In the above given example, the pair B-Q, which had at least two atoms, one from protein and another from RNA, with distance ≤3.40 Å, was considered as a positive pair. The pair A-Q, which had no atom–atom distance within the threshold, was considered as a negative pair. This procedure was applied to all 1336 RNA-protein complexes to identify the positive and negative pairs. Further, the peptides (protein with sequence length <25 amino acids) and small RNA (with sequence lengths <15 nucleotides) were excluded from these positive and negative data sets.

As a result, we obtained a training data set, namely, RPI1807, with 1807 positive pairs (consisting of 1807 protein and 1078 RNA chains) and 1436 negative pairs (with 1436 protein and 493 RNA chains). The positive and negative pairs of RPI1807 are shown in Supplementary Table S1.

### Test data sets

The RPI-Pred was tested with different data sets, including four data sets from previous studies and the new data set constructed in this work. The first three data sets were obtained from (36) and denoted as RPI369, RPI2241 and RPI13243 based on number of protein-RNA pairs (369, 2241 and 13 243), respectively. In (36), the first two data sets were used as training data sets to develop the classifier and the third was used to evaluate the classifier. RPI13243 consists of 13 243 RPI, which includes all 5166 protein-mRNA interactions published by Hogan *et al.* (49). The fourth data set (denoted as RPI367) consists of 367 protein-ncRNA interactions, constructed by Wang *et al.* (38) from the NPInter database (21).

The pairs in training data set RPI1807 were also used to construct the fifth test data set. In this case, the RPI-Pred was applied to predict RPI by using sequence and predicted structures for both protein and RNA, instead of the experimentally determined structures obtained from PDB (44). The sixth test data set was extracted from the NPInter database (21), namely, RPI10412, including 10 412 ncRPI pairs from six different model organisms. These ncRNA and protein pairs had been experimentally determined to have physical associations and listed in the ‘ncRBP’ category.

### PBs and RSS

In addition to primary sequences, we used structures, obtained from experimental determinations (available in PDB) or theoretical predictions, of both proteins and RNAs in our RPI-Pred. A protein 3D structure can be represented by 16-letter 1D structural fragments, called PBs (41). The PDB-2-PB database (50) provides the PB information based on the experimentally solved protein structures available in PDB (44). We used the PDB-2-PB to retrieve the 16-letter PB structure features for each protein in our training data set.

We used the 3DNA suite (51) to extract the RSS from the corresponding 3D structures (44). We used five category of RSS, namely, Stem (S), Hairpin (H), Loop (L), Bulges (B) and Internal loop (I) to construct our RPI-Pred method. In this study, the pseudo-knot RNA structures were not considered, because they were less numbers in our training data set. These PB and RSS were further combined with corresponding protein and RNA sequences to represent proteins and RNAs, respectively. These combined sequence-structure features were used to develop our RPI-Pred method for predicting potential ncRPI pairs.

### Representation of sequence and structural features

The sequence and structural features of protein and RNA used in this work were represented as follows. The protein sequence of 20 amino acids were classified into 7 groups (7-letter reduced sequence alphabets) according to their dipole moments and side chain volume: {A,G,V}, {I,L,F,P}, {Y,M,T,S}, {H,N,Q,W}, {R,K}, {D,E} and {C}. Then, we combined these 7-letter sequence features with the 16-letter PB structure representations, resulting in 112 (7 × 16) possible combinations. The normalized frequencies of 112 combinations formed the 112 protein vectors. Similarly, RNA sequence and RSS representations resulted in 4 × 5 possible combinations (4 for the nucleotide types; A, U, C and G and 5 for the RSS), and normalized frequencies of these 20 combinations resulted in 20 RNA vectors. The labels of sequence and structural features obtained from the proteins and RNA are given in Supplementary Table S2. In summary, to construct the RPI-Pred method, we used the sequence and structure features of 132 vectors, in which the first 112 vectors represented proteins and the remaining 20 vectors represented RNAs.

### SVM classifier

The SVM approach is a popular supervised machine learning technique used for many classification and regression problems (52). Here, we applied a well-known SVM classifier, LIBSVM-3.17 package (53) implemented as a standalone in-house program, to perform RPI prediction. We constructed our RPI-Pred method using 132-feature vectors that represent protein and RNA sequences and structures. RPI-Pred was optimized using different kernel functions with their corresponding parameters. We selected the ‘polynomial’ kernel function, which gave better prediction accuracy than others. RPI-Pred was trained to efficiently predict protein and RNA interaction pairs with the following optimized parameters: C = 1000, γ = 1, cofe0 = 1 and degree = 4.

### Predicting PBs and RSS

Since many proteins and RNAs have not been experimentally solved, we must use theoretical approaches to predict their structures. Many research groups have proposed PB prediction methods (42,54). In this work, we used the PB-kPRED method (55) to predict the PB structures for proteins included in all the test data sets.

Likewise, RSS can also be predicted with available RNA structure prediction methods (56–64). Here, we selected RNAfold from the Vienna package—an in-house standalone program (64) to predict the RSSs for RNAs in our test data sets. The predicted PB and RSS were combined with the corresponding amino acid and nucleotide sequences, respectively, and used in our RPI-Pred method.

### Performance evaluation

The performance of RPI-Pred was evaluated using 10-fold cross-validation (10-fold CV) approach. To perform this test, the training data set was divided into 10 subsets of equal size. Each subset was used for testing, while the remaining nine subsets were used for training. This process was repeated 10 times to cover all possibilities. Finally, we recorded the average performance over all 10 testing subsets. We evaluated the prediction performance by using Precision (PRE), Recall (REC), F-Score (FSC) and Accuracy (ACC), defined as follows:

\begin{equation*} Precision = \frac{{tp}}{{tp + fp}} \end{equation*}

\begin{equation*} Recall = \frac{{tp}}{{tp + fn}} \end{equation*}

\begin{equation*} F - Measure = \frac{{2*Precision*Recall}}{{Precision + Recall}} \end{equation*}

\begin{equation*} Accuracy = \frac{{tp + tn}}{{tp + fp + fn + tn}}*100 \end{equation*}

where, *tp* and *tn* denote the number of correctly predicted positive and negative pairs, respectively, and *fp* and *fn* denote the number of wrongly predicted positive and negative pairs, respectively. The area under curve (AUC) of the receiver operation characteristic curve was calculated using a 10-fold CV. The AUC ranges from 0 to 1, with 1 indicating the best prediction.

## RESULTS AND DISCUSSION

We built the RPI-Pred method for identifying binding partners of proteins or RNAs. In this section, we tested the performance of RPI-Pred on different test data sets, including RPI1807, RPI2241 and RPI369, and compared with previous methods. We also applied the RPI-Pred method to a large ncRPI data set and the predicted results were compared with other existing approaches.

### Performance of RPI-Pred with experimentally determined structures

The performance of our RPI-Pred method was evaluated using the 10-fold CV on RPI1807, RPI2241 and RPI369 data sets. The experimentally validated structures were extracted from PDB database (44). The performance of RPI-Pred was evaluated by calculating ACC, AUC, PRE, REC and FSC for each data set. Our RPI-Pred successfully predicted the RNA-protein pairs on RPI1807 data set with prediction accuracy (ACC) of 93%. The other measurements (AUC, PRE, REC and FSC) were observed as 0.97, 0.94, 0.95 and 0.95, respectively. The high prediction accuracy indicated that our method based on sequence and structure was reliably predicted RPI.

Similarly, the performance of RPI-Pred was evaluated using the positive and negative pairs of RPI2241 and RPI369 data sets. The positive pairs were directly adopted from RPI2241 and RPI369 data sets and their corresponding negative pairs were generated by following the steps reported in (38). Then, the RPI-Pred was applied on these data sets, to correctly predict all positive and negative pairs. The RPI-pred reached the prediction accuracy (ACC) of ∼84% for the RPI2241 data set. The AUC, PRE, REC and FSC were also observed as 0.89, 0.88, 0.78 and 0.83, respectively. Applying RPI-pred on the RPI369 data set resulted in a prediction accuracy of ∼92%, and AUC, PRE, REC and FSC of 0.95, 0.89, 0.89 and 0.89, respectively. The results in our newly constructed data set RPI1807 showed ∼10% and 2% increase in accuracy over RPI2241 and RPI369 results, respectively.

### Comparison of RPI-Pred with existing methods

We compared the performance of the RPI-Pred with Muppirala's method (36) on the RPI2241 and RPI369 data sets, respectively, using 10-fold CV. This comparison shows the prediction performance of the RPI-Pred method and the importance of structures in the prediction of RPI.

We compared our RPI-Pred results obtained from RPI2241 and RP1369 data sets (RPI2241-RPI-Pred and RPI369-RPI-Pred, respectively) using AUC, PRE, REC, FSC and ACC measurements. The comparison results are shown in Table 1. We denoted Muppirala *et al.*'s results as RPI2241-SVM, RPI369-SVM, RPI2241-RF and RPI369-RF, based on the two classifiers, SVM and RF, and the used training databases.

**Table 1.** Performance of RPI-Pred using 10-fold CV on RPI1807, RPI2241 and RPI369 data sets

Measurements	RPI2241			RPI369		
	RPI-Pred	RPISeq- SVM	RPISeq- RF	RPI-Pred	RPISeq- SVM	RPISeq- RF
AUC	0.89	0.97	0.92	0.95	0.81	0.81
PRE	0.88	0.87	0.89	0.89	0.73	0.75
REC	0.78	0.88	0.90	0.89	0.73	0.78
FSC	0.83	0.87	0.90	0.89	0.73	0.77
ACC	84.0	87.1	89.6	92.0	72.8	76.2

As shown in Table 1, our RPI2241-RPI-Pred result showed a prediction accuracy of 84%. This is ∼3% and ∼5% less than the results from the RPI2241-SVM (∼87%) and RPI2241-RF (∼89%) results, respectively. On the other hand, the RPI369-RPI-Pred result showed a prediction accuracy of 92%, implying an increase of ∼24%, and ∼18% over RPI369-SVM (∼72%) and RPI369-RF (∼76%) results, respectively.

These results illustrate that our RPI369-RPI-Pred could outperform RPI369-SVM and RPI369-RF classifiers in predicting the pairs of non-ribosomal RNA interacting with protein. Also inclusion of structure features can improve the RPI prediction (36). Slightly lower prediction accuracy was observed for the RPI2241 data set, which contained more ribosomal RNAs paired with proteins. Ribosomal RNA structures are more likely to contain pseudo-knot structures (40,65–67). However, the RNA-fold which was used in this work does not have the ability to predict such structures, and thus the proposed RPI-Pred cannot consider pseudo-knot structures. This may affect the correct prediction of ribosomal RNAs interacting with proteins. Further, Muppirala *et al.* (36) used 3-*mer* sequence features while our RPI-Pred uses 1-*mer* features of sequence and structure to perform RPI prediction. This leads to an increased dimensionality in feature space, which could lead to an improved prediction. However, this also results in a more complex model, and a significantly longer processing time.

Recently, Wang *et al.* (38) developed RPI prediction method using NB and ENB classifiers on RPI2241 and RPI369 data sets. We also compared the performance of our prediction of RPI-Pred method on these two data sets with Wang *et al.* (38) reported results. For this comparison, we grouped the results in (38) into four categories: RPI2241-NB, RPI369-NB, RPI2241-ENB and RPI369-ENB, based on the data set and classifiers used. The RPI-Pred method had an increased prediction accuracy of ∼9% and ∼10% over RPI2241-NB (75.7%) and RPI2241-ENB (74.0%), respectively. Our RPI-Pred method on RPI369 data set also showed an increased prediction accuracy of ∼15% and ∼17% over RPI369-NB (77.7%) and RPI369-ENB (75.0%), respectively.

### RPI-Pred performance with sequences and predicted structures

We further tested the RPI-Pred method by using sequences and predicted structures, instead of experimentally determined structures. This experiment was necessary due to the lack of experimentally validated structures for many RNAs, especially ncRNAs and proteins. The objective was to understand to what extent RPI-Pred performance might be affected by using predicted (rather than known) structures. To perform this analysis, we used the RPI-model constructed based on the RPI1807 data set and tested within the same data set. We observed a prediction accuracy of ∼83%. For the remaining measurements AUC, PRE, REC and FSC, the performance was 0.89, 0.79, 0.94 and 0.86, respectively. Compare with the results obtained using known structures as reported earlier (0.97, 0.94, 0.95 and 0.95 for AUC, PRE, REC and FSC, respectively). In particular, the prediction accuracy (ACC) decreased by nearly 10% compared with the performance of RPI-Pred on RPI1807 data set with known structures. We can observe similar decreases in the other performance measures. Expectedly, precision was significantly reduced when using predicted structures, while there was little or no impact on precision. Since, there is no experimental structural features were available for the rest of our test data sets (i.e. RPI367, RPI13243 and NPInter10412) we used the predicted PBs and RSS in order to perform the RPI prediction.

### Performance of RPI-Pred on predicting ncRPI pairs

Although most of the DNA transcripts are ncRNAs, very few have known functions. The ncRNA function can be predicted by identifying the different interacting partners, such as DNA, RNA and protein. It is currently believed that ncRNAs interact with proteins and then perform their regulatory functions, such as chromatin remodeling, to enhance or suppress gene expression (17–20). Therefore, studying ncRPI can reveal the importance of ncRNA in the post-transcriptional regulatory process. Very few computational studies (34–39) have been developed to predict the binding partner either for protein or ncRNA using both sequence and structural information. Here, we investigated the performance of our RPI-Pred in terms of predicting the binding partner for a given protein or ncRNA using both sequence and high-order structural information. We tested RPI-Pred method on RPI367, RPI13243 and NPInter10412 data sets, which contain ncRPI pairs. The results obtained from our RPI-Pred method for these three data sets were further compared with those results obtained by other exiting approaches.

Our RPI-Pred method was first tested using small RPI367 data set (38), consisting of 367 ncRPI pairs across six different model organisms: *Caenorhabditis elegans, Drosophila melanogaster, Escherichia coli, Homo sapiens, Mus musculus and Saccharomyces cerevisiae*. The RPI-Pred performances for the above six model organisms were given in Table 2. We compared the RPI-Pred prediction results with Wang *et al.*'s four classifiers (RPI369-NB (62%), RPI369-NB (77%), RPI2241-ENB (66%) and RPI2241-ENB (79%) results on the RPI367 data set. Our RPI-Pred method outperformed with a prediction accuracy of 89% (328 out of 367 pairs were correctly predicted), and none of Wang *et al.*'s classifiers performed at more than 80% accuracy (38). When compared prediction results from Wang *et al.*'s classifiers, RPI369-NB (62%), RPI369-NB (77%), RPI2241-ENB (66%) and RPI2241-ENB (79%), our RPI-Pred result showed greater improvement in prediction accuracy by 27%, 22%, 23% and 10%, respectively. Especially, our RPI-Pred predicted more ncRPI pairs (with the prediction accuracies of 100%, 92%, 96% and 91% for *C. elegans, D. melanogaster, E. coli* and *H. sapiens*) than each of Wang *et al.*'s classifiers.

**Table 2.** Comparison of RPI-Pred and Wang *et al.*'s classifiers (38) on the RPI367 data set

Organism	Total RNA-Protein pairs	RPI-Pred method (%)	RPI369-NB classifier (%)	RPI369-ENB classifier (%)	RP2241-NB classifier (%)	RP2241-ENB classifier (%)
*C. elegans*	3	3 (100%)	3 (100%)	1 (33%)	1 (33%)	1 (33%)
*D.melanogaster*	26	24 (92%)	13 (50%)	19 (74%)	23 (88%)	25 (96%)
*E. coli*	25	24 (96%)	13 (52%)	17 (68%)	12 (48%)	15 (60%)
*H. sapiens*	148	135 (91%)	93 (63%)	77 (52%)	84 (57%)	91 (62%)
*M. musculus*	46	37 (80%)	30 (65%)	40 (87%)	28 (61%)	37 (80%)
*S. cerevisiae*	119	105 (88%)	76 (64%)	89 (75%)	94 (79%)	118 (99%)
Total	367	328 (89%)	228 (62%)	243 (67%)	242 (66%)	287 (79%)

Our next ncRPI prediction analysis was performed on a larger data set used by Muppirala (36), which contains 13 243 ncRPI pairs. Our RPI-Pred method correctly predicted 12 240 out of 13 243 interaction pairs of this data set with the prediction accuracy of ∼92%. Our method showed ∼27% and ∼14% increases in accuracy, compared with Muppirala reported accuracies (65% and 78% with the SVM and RF classifiers, respectively).

Finally, we tested the ability of our RPI-Pred method to predict ncRPI pairs in the currently available NPInter database (version 2.0). NPInter database (21) is the only resource that provides the experimentally verified ncRPI pairs for different model organisms. Our new NPInter10412 data set consists of 10 412 ncRPI pairs of six model organisms from NPInter database. Since there are no experimentally validated negative pairs available in NPInter database, we randomly shuffled (i.e. by keeping the RNA fixed and reordered the proteins) all the positive pairs in NPInter10412 data set to make the negative data set. The performance of our RPI-Pred on NPInter10412 data set was evaluated by predicting correct positive and negative ncRPI pairs. The RPI-Pred had a prediction accuracy of ∼87% on NPInter10412 data set. The remaining measurements (PRE, REC and FSC) were observed as 0.85, 0.90 and 0.87, respectively.

Among the tested 10 412 positive ncRPI pairs, our RPI-Pred method correctly predicted 9335 interaction pairs with the accuracy of ∼90%. The RPI-Pred method predicted fewer ncRNA-protein pairs for *C. elegans, D. melanogaster* and *E. Coli* with the prediction accuracies of ∼78%, ∼77% and ∼76%, respectively. The RPI-Pred prediction accuracies for *H. sapiens, M. musculus* and *S. cerevisiae* were ∼89%, ∼97% and ∼82%, respectively. Table 3 shows the total number of positive ncRNA-protein pairs tested for each organism and the total number of pairs correctly predicted with our RPI-Pred method. The RPI-PRed prediction results in the NPInter10412 data set for each organism are shown in Supplementary Table S3.

**Table 3.** Performance of RPI-Pred on the NPIner10412 data set, for different organisms

Organism	Total ncRNA-protein pairs in NPInter10412	RPI-PRed performance (%)
*Caenorhabditis elegans*	36	28 (78%)
*Drosophila melanogaster*	91	70 (77%)
*Escherichia coli*	202	154 (76%)
*Homo sapiens*	6975	6193 (89%)
*Mus musculus*	2198	2147 (98%)
*Saccharomyces cerevisiae*	910	743 (81%)
Total	10 412	9335 (90%)

We further analyzed incorrect the prediction results of some specific complexes in each organism. We found a few cases in four organisms (*D. melanogaster, E. coli, H. sapiens* and *M. musculus*). Most of the cases, these incorrect predictions were observed for same protein that interacts with different ncRNAs. Among 21 false-negative ncRNA-protein pairsin *D. melanogaster*, 16 involved 3 proteins (Uniprot ID's: P17133, P26017 and Q9V3W7). Similarly, among 48 incorrect predictions in *E. coli*, 18 involved 3 proteins (P0AFZ3, P0C077 and P10121). The RPI-Pred method also failed to predict the pairs involving in two proteins (P19338 and P62312) in *H. Sapiens*. In the above few mentioned protein-RNA interactions, one protein interacts with multiple ncRNAs. This is due to wrongly predicted protein structures. In this proposed approach, our RPI-Pred method uses the features extracted from predicted structures of proteins and ncRNAs. Hence, our RPI-Pred prediction performance will be strongly influenced by the protein or RNA structure prediction approaches.

### Comparison of RPI-Pred with RPISeq for ncRPI prediction

Performance of our RPI-Pred method on NPInter10412 data set was further compared with the results obtained from existing approaches. We tested the NPIner10412 data set with RPISeq (36) and then the predicted results were compared with our RPI-Pred results. The standalone and locally implemented RPISeq program was obtained from the developers. Here, the RPISeq models, developed based on RF and SVM classifiers with RPI2241 and RPI369 data sets, were used to measure the RPI prediction performance on the NPIner10412 data set. To perform this test we used both the NPInter10412 positive pairs and the corresponding shuffled negative pairs. As previously reported (36), interactions with probability score ≥0.5 from RPISeq were considered as correct prediction. We further compared the predicted results of RPI2241-SVM, RPI369-SVM, RPI2241-RF and RPI369-RF models and the comparisons are given in Table 4.

**Table 4.** Comparison of RPI-Pred and RPISeq models (36) on the NPInter10412 data set

	RPI-Pred	RPI2241-RF	RPI2241- SVM	RPI369-RF	RPI369- SVM
PRE	0.85	0.50	0.50	0.43	0.42
REC	0.90	0.98	0.93	0.38	0.60
FSC	0.87	0.66	0.65	0.40	0.50
ACC	86.9	50.2	49.2	43.8	39.0

The prediction performance of the RPI2241-RF model on NPInter10412 data set was 0.50, 0.97, 0.66 and 0.50 for PRE, REC, FSC and ACC, respectively. The accuracy of RPISeq on NPInter10412 data set was just ∼50%. This performance is very low, when compared to our RPI-Pred accuracy ∼87%. However, RPI2241-RF correctly predicted more positive pairs as true positives (10 157 out of 10 412) and very few interactions were predicted as true negatives (288 out of 10 412). The other measurements PRE, REC and FSC were observed as 0.50, 0.98 and 0.66, respectively. We also observed the similar trend in the analysis of RPI2241-SVM results. The performance of RPI2241-SVM was ∼49% with more true positives (9682 out of 10 412 positive pairs were predicted) and fewer true negatives (730 out of 10 412 negative pairs were predicted). The other measurements PRE, REC and FSC were observed as 0.50, 0.93 and 0.65, respectively.

Similarly, we analyzed the results obtained by RPI369-RF and RPI369-SVM models. Table 4 shows that the RPI369-RF model predicted very few interactions as true positives (3972 out of 10 412), with nearly half of the interactions were predicted as true negatives (5125 out of 10 412). Therefore, the overall prediction accuracy was just ∼44%. The PRE, REC and FSC scores were observed at 0.43, 0.38 and 0.40, respectively. Similarly, theRPI369-SVM model had an overall prediction accuracy of only ∼39%. This model predicted more than half of the positive interactions as true positives (6271 out of 10 412) and many fewer negative interactions as true negatives (1851 out of 10 412). The remaining measurements PRE, REC and FSC were observed at 0.42, 0.60 and 0.50, respectively. The RPISeq prediction scores for each ncRNA-protein pair is given in Supplementary Table S3.

### Application of RPI-Pred for ncRNA-protein network construction

We further extended RPI-Pred method for *in silico* construction of ncRPI networks. Nacher and Araki (68) were among the first to study computational construction of ncRPI networks. They built interaction networks based on ncRPI of various model organisms, available in NPInter databases. Following the approach, Muppirala *et al.* (36) also used results from their proposed RPISeq method for the construction of ncRPI networks. Here, we extended our RPI-Pred approach to construct the the ncRPI networks to further study the important functions of ncRNAs. We evaluated our performance in predicting ncRPI in the NPInter database.

**Figure 2.**
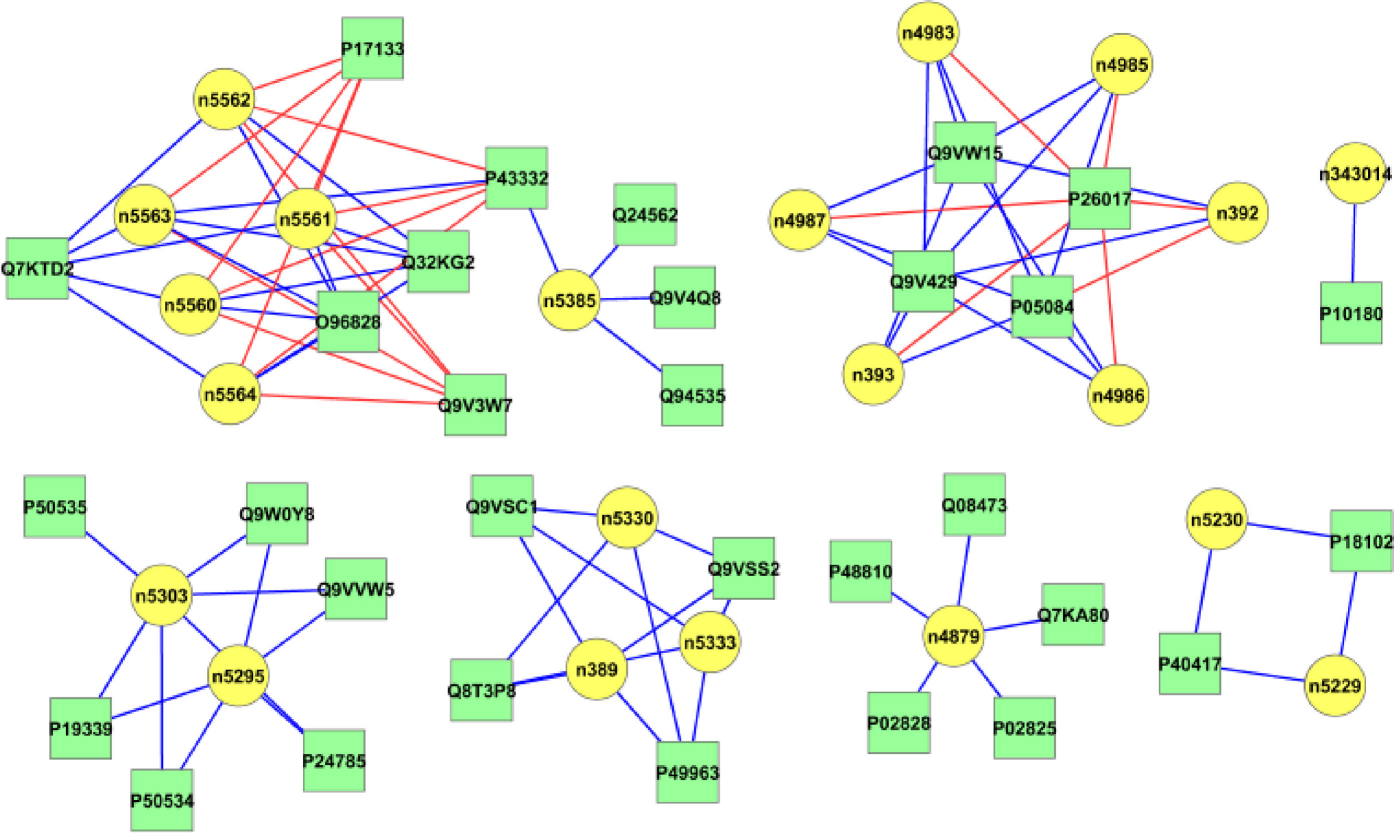
The ncRPI networks constructed based on interaction pairs predicted by RPI-Pred, for *D. melanogaster*. The ncRNA and proteins are shown in green (square) and yellow (oval/circular) nodes, respectively, while the correctly and wrongly predicted ncRPI are shown as blue and red edges, respectively.

In Figure 2, we show the interaction networks for 91 ncRPI of *D. melanogaster* that were obtained from NPInter database. Among the 91 positive interactions, the RPI-Pred method successfully predicted 70 interactions. The ncRPI of *D. melanogaster* contain both protein hubs (one protein interacting with multiple RNAs), and RNA hubs (one RNA interacting with multiple proteins). The *in silico*-based network construction helps to understand how many interactions were correctly predicted by our RPI-Pred method in the same protein or RNA hubs, and the reliability of our model in deriving new ncRPI and constructing new biological networks.

## CONCLUSION

Lots of the important fundamental cellular processes are medicated by protein and RNA interactions (RPIs); therefore, the study of RPI is valuable for the understanding of their functions. In the recent years, the high-throughput sequencing methods have led to the discovery of enormous amount of ncRNAs, which also interact with protein and regulate gene expression. Hence, it is very important to understand their function by studying the correct interaction partners. However, the experimental methods to determine correct interacting partner(s) for ncRNA are expensive and labor-intensive. In this case, computational approaches were highly relied to predict the interacting partner for ncRNA molecules. To our knowledge, very few studies have been reported for RPI prediction and none of the methods was considered the high-order protein and RNA structures, which are known to be vital to their functions.

In this work, we have developed a computational method, RPI-Pred, to address the prediction of RPI, and identification interacting partners of any given proteins or RNAs, using both sequences and structures of proteins and RNAs. Our proposed approach considered high-order structural features, namely, PBs and RSS, combined with their corresponding primary sequences for the investigation of RPI. Both experimental and predicted structures were used for RPI-Pred training and testing purposes. We tested the RPI-Pred method with a set of (nc)RPI data sets, and the results indicated that the proposed RPI-Pred was able to identify (nc)RPI with higher accuracy, when compared with other existing approaches. Therefore, our method is reliable to be applied to identify the binding partner(s) either for a protein or RNA. We further applied the method to *in silico* construction of ncRNA-protein networks. In addition, the proposed RPI-Pred method can also be extended to determine the binding partners (RNAs) for other types of proteins, such as transcription factors, which are able to interact with both DNA and RNA (69). A web server for the RPI-Pred can be freely accessed at http://ctsb.is.wfubmc.edu/projects/rpi-pred.

## SUPPLEMENTARY DATA

Supplementary Data are available at NAR Online.

SUPPLEMENTARY DATA
