# Supplementary material for: RPI-Pred: predicting ncRNA-protein interaction using sequence and structural information
Source: Nucleic Acids Res. 2015 Jan 21;43(3):1370–9. doi: 10.1093/nar/gkv020 (PMC4330382; doi:10.1093/nar/gkv020)
Supplement: SUPPLEMENTARY DATA [file supp_43_3_1370__index.html]

RPI-Pred: predicting ncRNA-protein interaction using sequence and structural information — SUPPLEMENTARY DATA 

# RPI-Pred: predicting ncRNA-protein interaction using sequence and structural information

## SUPPLEMENTARY DATA

**Files in this Data Supplement:**

- Supplementary Tables Captions
- Table S1
- Table S2
- Table S3
