# Supplementary material for: RPI-Pred: predicting ncRNA-protein interaction using sequence and structural information
Source: Nucleic Acids Res. 2015 Jan 21;43(3):1370–9. doi: 10.1093/nar/gkv020 (PMC4330382; doi:10.1093/nar/gkv020)
Supplement: SUPPLEMENTARY DATA [file supp_gkv020_nar-03010-z-2014-File004.docx]

**Supplemental data:**

Table S1: Training dataset (RNA-protein positive and negative pairs)

Table S2: The sequence and structural feature labels for protein and RNA

Table S3: The RPI-Pred prediction results compared with RPISeq – all four models (30) on NPInter10412 dataset
