# Supplementary material for: RPI-Pred: predicting ncRNA-protein interaction using sequence and structural information
Source: Nucleic Acids Res. 2015 Jan 21;43(3):1370–9. doi: 10.1093/nar/gkv020 (PMC4330382; doi:10.1093/nar/gkv020)
Supplement: SUPPLEMENTARY DATA [file supp_gkv020_nar-03010-z-2014-File006.docx]

**Table S2:**

Protein sequence and structural feature labels

| **Sequence features label** | **Amino acid representation** | **Structure features label** | **Secondary structure representation** |
| --- | --- | --- | --- |
| a | {A, G, V} | A, B, C | N – cap β strand |
| b | {I, L, F, P} | D | β strand |
| c | { Y, M, T, S} | E, F | C – cap β strand |
| d | {H, N, Q, W} | G, H, I, J | Coil |
| e | {R, K} | K, L | N – cap α helix |
| f | {D, E} | M | α helix |
| g | {C} | N, O, P | C – cap α helix |

RNA sequence and structural feature labels

| **Sequence features label** | **nucleotide representation** | **Structure features label** | **Secondary structure representation** |
| --- | --- | --- | --- |
| A | {A} | H | Helices |
| D | {D} | N | Hairpin loops |
| G | {G} | B | Bulges |
| C | {C} | I | Internal loops |
|  |  | L | loops |
